# The SToICAL trial: study protocol for the soft tissue injection of corticosteroid and local anaesthetic trial—a single site, non-inferiority randomised control trial evaluating pain after soft tissue corticosteroid injections with and without local anaesthetic

**DOI:** 10.1186/s13063-021-05627-5

**Published:** 2021-09-28

**Authors:** M. Jones, J. Evans, S. Fullilove, E. Doyle, C. Gozzard

**Affiliations:** 1 Trauma and Orthopaedic Department, University Hospitals Plymouth NHS Trust, Plymouth, PL6 8DH UK; 2 Health Services and Policy Research Group, University of Exeter, St Lukes Campus, 79 Heavitree Road, Exeter, EX1 1TX UK

**Keywords:** Corticosteroid, Local anaesthetic, Trigger Finger, De Quervains tenosynovitis, Carpal tunnel syndrome, Visual analogue scale

## Abstract

**Background:**

Corticosteroid injections are used in the treatment of hand and wrist conditions. The co-administration of a local anaesthetic and corticosteroid aims to reduce pain after the injection, although no studies have directly compared this with using corticosteroid alone. The aim is to determine whether pain experienced during the 24 h after a corticosteroid injection to the hand and wrist is no worse than (not inferior to) the pain experienced after a corticosteroid and local anaesthetic injection.

**Methods:**

A single-site, patient- and assessor-blinded, non-inferiority randomised control trial recording pain visual analogue scale (VAS) scores in patients with a clinical diagnosis of trigger finger, de Quervains tenosynovitis or carpal tunnel syndrome, treated with a 1-ml triamcinolone (40 mg/1 ml) injection co-administered with or without 1 ml of 1% lidocaine. The primary aim is to investigate a difference in pain VAS scores at 1 h after the injection using a mean change score.

A 95% power calculation was made using a minimally clinical important difference of 20 mm as the clinically admissible margin of non-inferiority and an assumed standard deviation of 25 mm, from previous studies. Including a 20% fall out rate, 100 patients are required.

**Discussion:**

Patients with a clinical diagnosis of trigger finger, de Quervains and carpal tunnel syndrome, are over the age 18 years old and who are able to give written informed consent will be included. Patients will be excluded if they have had previous surgery or corticosteroid injection for the condition being treated at the site considered for injection.

Patients will be electronically randomised and injections delivered during their clinic appointment. Pain is assessed using a 100-mm VAS score taken, before and at the time of injection and at 5 min, 1 h, 2 h, 3 h and 24 h after the injection. The secondary outcomes are to determine a difference in pain VAS score at the time of injection and during the 24 h after.

**Trial registration:**

This study is registered on the IRAS (259336) on November 11, 2019, and EudraCT database on October 31, 2019 (2019-003742-32). REC/HRA approval was given in January 2020, and Clinical Trial Authorisation from the MHRA was given in December 2019. The study is registered on ClinicalTrials.gov (NCT04253457) on February 5, 2020.

**Supplementary Information:**

The online version contains supplementary material available at 10.1186/s13063-021-05627-5.

## Administrative information

*Title:*

The SToICAL Trial: Study protocol for The Soft Tissue Injection of Corticosteroid And Local aneasthetic trial – A single site, non-inferiority randomised control trial evaluating pain after soft tissue corticosteroid injections with and without local anaesthetic

*Trial registration:*

ClinicalTrials.gov Identifier: NCT04253457

*Protocol version:*

Version 2 (Final) – 7 January 2020

*Funding:*

This study will be funded from the research budget held by Trauma and Orthopaedic Department at the University Hospital Plymouth NHS Trust, and is not commercially funded.

*Role and responsibilities:*

Lead Co-Author and Principle Investigator:

Mr M Jones

Trauma and Orthopaedic Department

University Hospitals Plymouth NHS Trust

PL6 8DH

m.jones16@nhs.net

Lead Co-Author:

Mr J Evans*

NIHR Clinical Lecturer - University of Exeter

Health Services and Policy research Group

St Lukes Campus

79 Heavitree Road

Exeter

EX1 1TX

j.p.evans@exeter.ac.uk

Chief Investigator:

Mr C Gozzard

Trauma and Orthopaedic Department

University Hospitals Plymouth NHS Trust

PL6 8DH

charlesgozzard@nhs.net

Co-investigators:

Miss S Fullilove

Trauma and Orthopaedic Department

University Hospitals Plymouth NHS Trust

PL6 8DH

sue.fullilove@nhs.net

Co-investigators:

Dr E Doyle

Trauma and Orthopaedic Department

University Hospitals Plymouth NHS Trust

PL6 8DH

edoyle@nhs.net

*Trial Sponsor:*

Corinna Mossop

Research Development and Innovation (RD&I)

University Hospital Plymouth NHS Trust

The Research Office

Level 2 MSCP

Bircham Park Office

1 Roscoff Rise

Derriford

Plymouth

*Trial Steering Committee:*

Independent Chairman

Mr Jon Keenan

Trauma and Orthopaedic Department

University Hospitals Plymouth NHS Trust

jonathan.keenan@nhs.net

Mr C Gozzard

Trauma and Orthopaedic Department

University Hospitals Plymouth NHS Trust

charlesgozzard@nhs.net

Independent Expert

Dr M Chopra

Consultant in Paediatric Anaesthesia

University Hospitals Plymouth NHS Trust

michellechopra@nhs.net

Pharmacy Representative

Mark Marner

Pharmacy Clinical Trials Support Manager

University Hospitals Plymouth NHS Trust

mike.marner@nhs.net

Chief Investigator

Mr C Gozzard

Trauma and Orthopaedic Department

University Hospitals Plymouth NHS Trust

charlesgozzard@nhs.net

Principle Investigator

Mr M Jones

Trauma and Orthopaedic Department

University Hospitals Plymouth NHS Trust

PL6 8DH

m.jones16@nhs.net

Sponsor Representative

Victoria Yates

Senior Research Support Facilitator

University Hospitals Plymouth NHS Trust

victoriayates@nhs.net

## Introduction

### Background

Corticosteroid injections are used in the treatment of a variety of hand and wrist conditions. The local anti-inflammatory effect they impart is believed to be due to downregulation of the cytokine pathway [1]. The injections can be administered intra or extra-articular, with or without image guidance and with or without local anaesthetic. The co-administration of a local anaesthetic and corticosteroid is commonly undertaken and aims to reduce the procedural pain associated with the injection itself. However, no studies have directly compared the patient’s perception of procedural pain in corticosteroid injection that include or omit local anaesthetic.

Trigger finger, de Quervains tenosynovitis and carpal tunnel syndrome are all painful conditions of the hand and wrist where corticosteroid injections have a role in their treatment. Trigger finger is a condition that causes locking on flexion of the involved finger, dysfunction and pain as a result of thickening of the first annular pulley [2, 3]. Corticosteroid injection is an effective treatment with cure rates ranging from 60 to 92% [3]. De Quervains tenosynovitis is a disorder that causes radial sided wrist pain as a result of mechanical impingement of the tendons within the first extensor compartment [4]. Corticosteroid injections have a reported cure rate of 83% [5]. Carpal tunnel syndrome causes pain and numbness in a median nerve distribution in the hand from compression of the nerve within the carpal tunnel. Corticosteroid injection is used in mild and moderate cases; however, surgery is preferred in severe cases [6].

Corticosteroid injections administered into soft tissue for these conditions can be performed under ultrasound guidance or by using anatomical landmarks. A cadaveric study showed no statistical difference in the accuracy between the two methods of injecting for de Quervains tenosynovitis or carpal tunnel syndrome [7]. No superior clinical benefit was found when using ultrasound for trigger finger corticosteroid injections, and the extra time and cost required was noted [8]. Landmark-guided corticosteroid injections for these conditions are performed routinely in clinical practice across the National Health Service (NHS) in the UK. Evidence supports the efficacy for landmark-guided injections; ultrasound guidance has not been reported to be superior and requires additional cost, has a delay in treatment, is a secondary appointment, and is user dependent [9, 10].

Corticosteroids are not immediately effective; it is often 3 to 7 days before their therapeutic benefit is felt [11]. Corticosteroids are therefore often mixed with local anaesthetic and co-administered under the assumption that these reduce the initial procedural pain of the injection. The mixing of these substrates does not affect the efficacy of the corticosteroid and is known not to alter the propensity for corticosteroid crystals to aggregate or change size. They are therefore deemed safe to co-administer [12].

Lidocaine is the most commonly administered local anaesthetic for this indication and has a rapid onset of less than 2 min, and its analgesic effects can last for 1 to 3 h [13]. It is, however, notable that an injection containing lidocaine can itself be painful because of the acidity of the solution. The acidity can be neutralised with sodium bicarbonate. However, a randomised control trial failed to demonstrate a statistically significant benefit of sodium bicarbonate in extra-articular corticosteroid injections with local anaesthetic to reduce post procedural pain in the hand and wrist [9].

### Rationale

The authors are not aware that a study exists that assesses pain immediately following soft tissue corticosteroid injections in the hand and wrist, co-administered with and without local anaesthetic. Although corticosteroids and local anaesthetic are often co-administered, no evidence is currently available to support this.

The effects of the local anaesthetic only last for 2–3 h; therefore, it would not provide relief from post-procedure corticosteroid flare nor bridge the time until the corticosteroid became effective. A post injection flare is an increase in pain that results from a local increase in inflammation that develops within hours and can last 2–3 days [14]. Administering local anaesthetic can be painful because of the acidity of the solution and, as a consequence of their co-administration, higher volume injections are given.

Soft tissue injections of corticosteroid in the hand are widely used by health professionals across different specialities; therefore, the potential implications of the trial are far reaching. A definitive outcome would help minimise variation in practice. If post injection pain with administering corticosteroid alone is not inferior to co-administering it with local anaesthetic, this could stop unnecessary medication being given to patients. This would reduce any potential temporary numbness which may result from injecting local anaesthetic and may prevent patients from driving home after their appointment. Adding local anaesthetic also increases the cost, time and clinical waste of the treatment. Although the additional cost per treatment may not be significant when considering the number performed across the National Health Service, the saving may be considerable. However, any findings will only be relevant for the specific procedures studied and may not be applicable to other procedures where corticosteroid and local anaesthetic are co-administered.

### Hypothesis

We hypothesise that the pain experienced at 1 h after a corticosteroid injection for trigger finger, de Quervains tenosynovitis and carpal tunnel syndrome is no worse than when the corticosteroid is co-administered with local anaesthetic.

### Trial design

This is a single-site, patient- and assessor-blinded, non-inferiority randomised control trial where patients will be randomised with a 1:1 allocation ratio to receive an injection of corticosteroid alone or an injection of corticosteroid co-administered with local anaesthetic to treat, trigger finger, de Quervains tenosynovitis or carpal tunnel syndrome.

## Methods

### Study setting

This study will be set in the Orthopaedic Outpatient clinic at a single University Hospital in the UK, UHPT.

### Eligibility criteria

The reviewing physician will approach potentially eligible patients within the Outpatient Clinic.

### Inclusion criteria

- Male or female ages ≥ 18 years old
- A clinical diagnosis of trigger finger, de Quervains tenosynovitis or carpal tunnel syndrome made by a consultant physician
- Treatment with corticosteroid injection is recommended by the consultant physician and agreed by the patient
- Patient is willing and able to give informed consent for participation in the study

### Exclusion criteria

- Previous surgery for the condition being treated at the desired location of injection*
- Previous steroid injection for the condition being treated at the desired location of injection*
- Clinical suspicion of local or systematic sepsis or infection
- History of hypersensitivity to the corticosteroid or local anaesthetic
- Pregnant or breast-feeding females
- Unable to understand and complete self-report questionnaires written in English
  *Previous surgery or a corticosteroid injection elsewhere in the hand or wrist does not exclude the patient from the trial

### Interventions

There are a variety of injectable synthetic corticosteroids with different half-lives and variable duration of clinical benefit [15]. Triamcinolone (40 mg/1 ml) is used routinely for soft tissue corticosteroid injections of the hand and wrist at the University Hospitals Plymouth NHS Trust (UPHT) by the local Hand and Wrist surgeons.

Study participants will receive a single injection containing one of the two following solutions, delivered under landmarks guidance by a physician to treat either trigger finger, de Quervains tenosynovitis or carpal tunnel syndrome:

A. 1 ml of triamcinolone (40 mg/1 ml)
  or
B. 1 ml of triamcinolone (40 mg/1 ml) + 1 ml 1% lidocaine

### Outcomes

This trials’ primary outcome is a difference in pain visual analogue scale (VAS) scores at 1 h after a corticosteroid injection for trigger finger, de Quervains tenosynovitis or carpal tunnel syndrome co-administered with or without local anaesthetic.

The secondary outcomes include:

i) The difference in pain VAS scores during the 24 h after injection (co-administered with or without local anaesthetic)
ii) The difference in the pain VAS scores at the time of the injection (co-administered with or without local anaesthetic)
iii) The difference in the additional analgesia required and in the functional use of the hand during the first 3 h following an injection (co-administered with or without local anaesthetic)

### Participant timeline

**Fig. 1:**
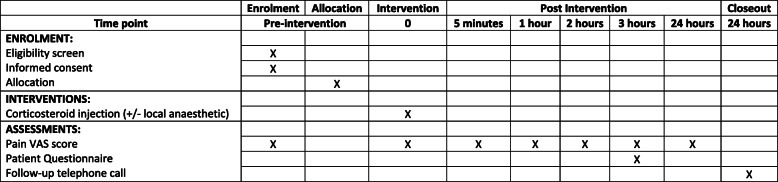
Summary of Investigations, treatments and assessments

Pain is subjective and multi-dimensional; therefore, it is difficult to fully evaluate the complete pain experience. The 100-mm pain VAS score is a widely used method for assessing pain. Pain will be assessed using this method, prior to any intervention, during the intervention, and then 5 min, 1 h, 2 h, 3 h and 24 h following the intervention (Fig. 1).

### Sample size

As part of the literature review, it was identified that a 20-mm MCID on a 100-mm scale has been utilised in other studies evaluating post procedural pain [16], one of which was also investigating pain following soft tissue corticosteroid injections in the hand [14]. The authors are not aware of any studies that have specifically evaluated and set a standardised minimally clinical important difference (MCID) in pain VAS score following soft tissue corticosteroid injection in the hand and wrist. Although this is recognised as a limitation, it was felt that the ubiquitousness of the VAS as previously validated tool in multimodal and multisite pain, and its previously demonstrated utility and patient comprehension meant that it can be regarded as the most appropriate patient-reported pain score.

A MCID of 20 mm will be used as the clinically admissible margin of non-inferiority. From previous studies, a standard deviation of 25 mm and with a 95% power calculations have determined a required sample size of 41 per study arm, when including a 20% fall out rate a total sample size of 100 patients will be required [14]. All sample size calculations were conducted at the two-sided 5% significance level and 95% CIs with Stata 14 (StataCorp. 2015. Stata Statistical Software: Release 14. College Station, TX: StataCorp LP).

### Recruitment

**Fig. 2:**
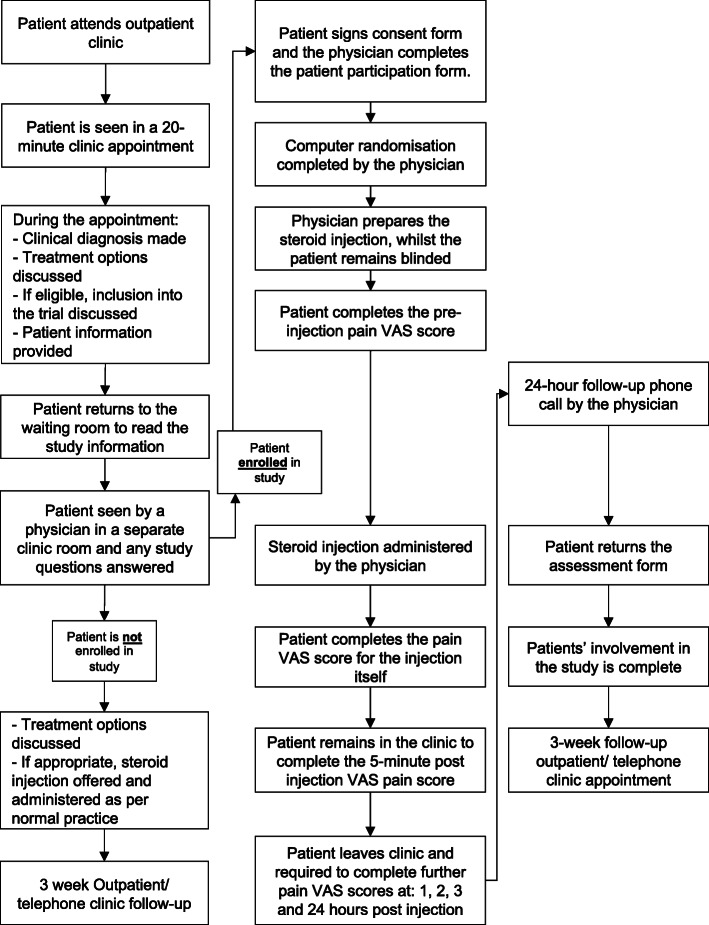
Patient recruitment process during outpatient clinic

Potentially eligible patients will be identified from referral letters and approached prior to their clinic appointment. Patients considering enrolment will be given sufficient time to go through the study information at their own pace (Fig. 2). A prospective audit was conducted demonstrating that an average of 3.6 corticosteroid injections was performed per week and an estimated 60% capture rate; full recruitment should be achievable within 12 months.

### Allocation

Patients will be randomised by the recruiting physician using the simple randomisation service provided by Sealed Envelope Ltd. [17]. This electronic online service will provide random permuted block randomisation, ensuring the participants are balanced between the control and treatment group.

### Blinding

The patient, assessor and data analysts will be blinded to the assigned treatments. The physician will perform the randomisation, and then draw up and prepare the intervention in a separate room away from the patient. Neither treatment can be distinguished from one another by appearance alone. Returned patient assessment records which will include all pain VAS scores will remain sealed until recruitment is complete. The pain VAS scores will then be measured, recorded on and statistically analysed by members of the study management team, who were not involved with patient recruitment or treatment.

In the instance of an adverse reaction or serious adverse reaction, it would be permissible to unblind the patient to aid the reporting and investigation of such event.

### Data collection methods

Patients recruited to the study will complete a consent form with someone registered on the delegation log with appropriate training (Additional file 1). A patient participation record will be completed by the physician (Additional file 2). A photocopy of the consent form will be given to the patient and another copy kept in the patients’ hospital notes. The original consent form will be kept in the site file along with the patients’ participation record. The patient participation record contains a patient participation number, which is the only link between the patient and the treatment delivered.

Prior to any intervention, the patient will complete a pain VAS score, on a 100-mm scale on a paper form, as a baseline from which change scores can be calculated. During the clinic appointment, further pain VAS scores are completed to record the pain experience at the time of the injection and at 5 min post injection. The pain VAS score of the pain experience during the corticosteroid injection will be recorded immediately after the procedure. The patient then takes an assessment record home to complete further pain VAS scores at 1 h, 2 h, 3 h and 24 h following the injection. The timings at which these scores must be recorded will be clearly documented on the patient assessment record by the physician before leaving clinic as a reminder. A potential limitation of the recording process is that the responsibility will be left with the patient to record the score at the desired time points. A clearly structured plan will be provided on discharge outlining these time points and the importance of the intervals between them. There are three further questions, on the same form, that the patient will be required to answer at 3 h post injection.

During the first 3 h after your steroid injection:

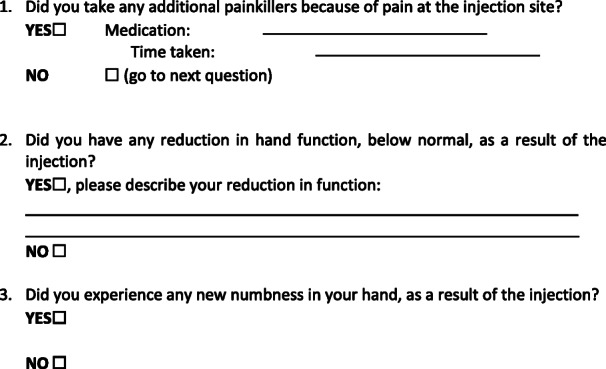



After the final pain VAS score at 24-h, the patient will receive a follow-up call by the physician to ensure the assessment form has been completed, to answer any questions and remind the patient to return the form using the prepaid envelope provided. After the form is posted, the patients’ participation in the study will be complete. Participants who have not returned their patient assessment records will not be included in the analysis. Results from partially completed forms will be included for analysis.

### Data management

The participants’ data will be pseudonymised (i.e. only initials and a study ID number on the patient’s participant record and any electronic database will identify participants). All documents will be stored securely and only be accessible by trial staff and authorised personnel. The study will comply with the Data Protection Act 2018. No participants will be individually identified in any subsequent publications relating to this study. Archiving will be authorised by the sponsor following submission of the end of study report. All essential documents will be archived for a minimum of 5 years after completion of trial. Destruction of essential documents will require authorisation from the study sponsor.

### Statistical methods

The primary objective of investigating a difference in pain VAS scores at 1 h after a corticosteroid injection will primarily be determined by analysing the mean change in pain VAC score between 1-h post injection and baseline pre-injection will be performed. The primary intention will be the use of parametric tests (unpaired Student’s *T* test) dependent upon the assessment of score distribution (skewness and kurtosis). Non-parametric testing will be employed if the mean distribution fails tests of normality. Secondary outcome measures of the primary objective will involve determining the effect size at 1 h post injection to measure significance of the difference between the study and control group.

Secondary outcome analysis will also be conducted to investigate whether there is a difference in pain VAS scores during the 24 h. The cumulative pain in the 24-h period in both groups will be plotted and the total area under the curve (AUC) will be calculated. Multilevel linear regression analysis will be conducted to compare 24-h pain VAS with injection intervention nested in patient condition.

Sensitivity analysis will be conducted to investigate whether there is a difference in the pain VAS scores at the time of the injection by using a multilevel linear regression analysis to compare pain VAS scores with injection intervention nested in the patient condition.

The percentage of patients requiring additional analgesia or who experienced worsening hand function will be determined from the three questions asked in the patient assessment record. The results will be presented as numbers with additional details given in the patients’ response, but no statistical analysis will be performed on these results. No subgroup analysis will be performed.

### Data monitoring

The data monitoring committee will monitor the return rate for the patient assessment record. These records will remain sealed until recruitment is complete, so no interim analysis of outcome data or compliance will be performed. All adverse events will be record and reported, depending on the nature of the event. There are no additional provisions for trial patients beyond the normal standard of care as the trial compares two treatments routinely used in normal practice.

## Ethics and dissemination

### Research ethics approval

This study has been reviewed by the South West – Central Bristol Research Ethics Committee and received a favourable opinion. In accordance with approval conditions, the study has been registered on ClinicalTrials.gov (Identifier: NCT04253457).

### Protocol amendments

Any amendments to the study protocol will be reviewed by the Health Research Authority and, if required, the Research and Ethics Committee and will not be implemented until a favourable opinion is granted. Amendments will then be reviewed and accepted by the NHS Research and Development department before they can be implemented in practice.

### Dissemination policy

It is proposed that the study team will prepare a plain English summary of the study results, which will be sent to the study participants as soon as possible after the end of the study. The final results of the study will be disseminated via presentations at appropriate scientific meetings and conferences and publication in appropriate peer-reviewed journals. The final dataset will be made available to on the trial delegation log and anonymised patient level date submitted as an appendix with any subsequent publication submission.

### Funding

This study will be funded by available department research funds, held by the Research and Development department, and is not commercially funded.

## Discussion

Findings for the proposed study are expected to benefit both primary and secondary care settings involved in the administration of soft tissue corticosteroid injections into the hand.

Despite the large number of corticosteroid injections administered in the National Health Service (NHS) and the variability in practice, there is no published data on the pain following soft tissue corticosteroid injections in the hand and wrist, co-administered with and without local anaesthetic.

Co-administering corticosteroid and local anaesthetic in an injection should not improve the pain experienced from the injection itself. The effects of the local anaesthetic are short lived and will not provide relief from any post-procedure corticosteroid flare nor bridge the time until the corticosteroid became effective.

Adding local anaesthetic may potentially make the injection more painful because of the acidity of the solution and the higher volume administered. In addition, the use of local anaesthetic may even worsen paraesthesia symptoms or result in paraesthesia in parts of the hand, preventing normal function for the duration of its action.

It is also notable that adding local anaesthetic increases the cost, time and clinical waste of the treatment. Although the additional cost per treatment may not be significant, when considering the number performed across the NHS, the saving may be considerable.

## Trial status

Protocol version 2 (final), 7 January 2020. Recruitment will commence in November 2020 and is anticipated to be completed by November 2021.

## Supplementary Information

**Additional file 1.** Participant consent form.

**Additional file 2.** Patient participation record.

## Abbreviations

VAS: Visual analogue scale
UHPT: University Hospital Plymouth NHS Trust
MCID: Minimally clinical important difference
NHS: National Health Service
