# Supplementary material for: The SToICAL trial: study protocol for the soft tissue injection of corticosteroid and local anaesthetic trial—a single site, non-inferiority randomised control trial evaluating pain after soft tissue corticosteroid injections with and without local anaesthetic
Source: Trials. 2021 Sep 28;22:662. doi: 10.1186/s13063-021-05627-5 (PMC8479928; doi:10.1186/s13063-021-05627-5)
Supplement: Supplementary file 1 — Additional file 1. Participant consent form. [file 13063_2021_5627_MOESM1_ESM.pdf]

NHS
University Hospitals
Plymouth
NHS Trust

PARTICIPANT CONSENT FORM

The SToICAL Study – The Soft Tissue Injection of Corticosteroid And Local anaesthetic

Name of Researcher: Mr C Gozzard/ Miss S Fullilove/ Dr E Doyle/ Mr M Jones/ Mr J Evans

Patient participation number: ☐☐☐☐☐☐☐

Study treatment number: ☐☐☐☐☐☐☐

Please initial all boxes

1. I confirm that I have read and understand the patient information sheet dated………… (version ………….) for the above study. I have had the opportunity to consider the information, ask questions and have had these answered satisfactorily. ☐

2. I understand that my participation is voluntary and that I am free to withdraw at any time without giving any reason, without my medical care or legal rights being affected. ☐

3. I understand that relevant sections of my medical notes and data collected during the study, may be looked at by individuals from regulatory authorities or from the NHS Trust, where it is relevant to my taking part in this research. I give permission for these individuals to have access to my records. ☐

4. I agree to be contacted by a member of the study team as part of the study follow-up the day after my steroid injection ☐

5. I agree to my GP being informed of my participation in the study. ☐

6. I agree to take part in the above study. ☐

7. I would like to be informed of the results of the study, when they are available and agree that my contact details can be retained for this purpose. ☐

____________________	____________________	____________________
Name of Participant	Date	Signature
____________________	____________________	____________________
Name of Person taking consent	Date	Signature

When completed: 1 copy for participant; 1 copy in medical notes, 1 copy provided to the sponsor and original copy for researcher site file.

IRAS No:259336 version 2 (final), date 7 Jan 2020
