# Supplementary material for: The SToICAL trial: study protocol for the soft tissue injection of corticosteroid and local anaesthetic trial—a single site, non-inferiority randomised control trial evaluating pain after soft tissue corticosteroid injections with and without local anaesthetic
Source: Trials. 2021 Sep 28;22:662. doi: 10.1186/s13063-021-05627-5 (PMC8479928; doi:10.1186/s13063-021-05627-5)
Supplement: Supplementary file 2 — Additional file 2. Patient participation record. [file 13063_2021_5627_MOESM2_ESM.pdf]

PATIENT PARTICIPATION RECORD

This participation record must be completed prior to patient participation and delivery of treatment. This record will be stored in the study folder at the study site until the completion of the trial.

Eligibility screening summary:

Inclusion Criteria

The following criteria MUST all be answered YES for the patient to be included

		Yes	No
1.	Male or Female aged 18 years or over		
2.	A Clinical diagnosis of trigger finger, de Quervains tenosynovitis, or carpal tunnel syndrome		
3.	Patient is willing and able to give informed consent for participation in the study		
4.	Treatment with corticosteroid injection is recommended by the doctor and agreed by the patient		

Exclusion Criteria

The following criteria MUST all be answered NO for the patient to be included

		Yes	No
1.	Previous surgery for the condition being treated at the desired location of injection		
2.	Previous steroid injection for the condition being treated at the desired location of injection		
3.	Clinical suspicion of local or systematic sepsis or infection		
4.	History of hypersensitivity to the corticosteroid or local anaesthetic		
5.	Pregnant or breast-feeding females		
6.	Unable to understand and complete self-report questionnaires written in English		

Patient Demographics:

First name: ______________________________

Surname: ______________________________

Hospital Number: ☐☐☐☐☐☐☐

DOB: ☐☐/☐☐/☐☐

Contact number: ______________________________

Sex: Male ☐ Female ☐

Study checklist:

Patient information provided ☐

Patient consented ☐

Patient randomised ☐

Randomisation process:
To randomise a patient, go to the following website and use the password below.

Go to: https://www.sealedenvelope.com/simple-randomiser/v1/

Trial name: SToICAL Study

Password: Stocial2020

Patients will be randomised to group A (Treatment) or B (Control). Draw-up the treatment as stated below. Then write A/ B in the study treatment box.

Patient participation number: ☐☐☐☐☐☐☐

Study treatment: ☐

Treatments:

A. 1ml of triamcinolone (40mg/1ml)

or

B. 1ml of triamcinolone (40mg/1ml) + 1ml 1% lidocaine

Treatment for:

Carpal tunnel syndrome   Trigger finger   De Quervains

Physician name: ____________________________________

Physician Signature: ____________________________________

Date of treatment: ☐☐/☐☐/☐☐
